# Effect of drying processes on the occurrence of lipid oxidation‐derived 4‐hydroxy‐2‐hexenal and 4‐hydroxy‐2‐nonenal in Spanish mackerel (*Scomberomorus niphonius*)

**DOI:** 10.1002/fsn3.3138

**Published:** 2022-11-15

**Authors:** Kexin Cui, Nan Liu, Yong Sun, Guohui Sun, Shanshan Wang, Min Yang, Xiaoli Wang, Deqing Zhou, Yinggang Ge, Dajun Wang, Mingli Wang

**Affiliations:** ^1^ Chinese Academy of Fishery Sciences Yellow Sea Fisheries Research Institute Qingdao China; ^2^ College of Food Science and Engineering Ocean University of China Qingdao China; ^3^ Yantai Haiyu Food Co., Ltd. Yantai China; ^4^ Penglai Huiyang Food Co., Ltd. Yantai China

**Keywords:** 4‐hydroxy‐2‐hexenal, 4‐hydroxy‐2‐nonenal, dry‐cured spanish mackerel, lipid oxidation, occurrence

## Abstract

In this study, dry‐cured Spanish mackerel (*Scomberomorus niphonius*, DCSM) was prepared via three different methods (hot‐air drying, cold‐air drying, and sun drying). The content of 4‐hydroxy‐2‐hexenal (HHE) and 4‐hydroxy‐2‐nonenal (HNE) derived from lipid oxidation in whole processes was investigated by HPLC‐MS/MS. The changes in fatty acid composition were detected by GC‐MS, and the degree of lipid oxidation was evaluated by the levels of acid values (AV), peroxide values (POV), and thiobarbituric acid‐reactive substances (TBARS). The results showed that the drying process significantly accelerated lipid oxidation in DCSM. The contents of HHE and HNE were significantly increased after processing. The content of HHE was higher by 18.44‐, 13.45‐, and 16.32‐folds compared with that of HNE after three different processes, respectively. The HHE and HNE contents fluctuated upward during the hot‐air and cold‐air drying process. However, the contents of HHE and HNE increased time‐dependent during the sun drying process, with the highest values of 86.33 ± 10.54 and 5.29 ± 0.54 mg/kg fish among the three different processes. Besides, there was a significant positive correlation between HHE contents and n‐3 fatty acids content in hot‐air drying and sun drying processes (Pearson's *r* = .991/.996), and HNE occurrence was closely related to n‐6 fatty acid content in sun drying process (Pearson's *r* = .989). Regression analysis indicated that the content of HHE and TOTOX_TBA_ values in DCSM showed good linear relationships (*R* ^2^ value = .907), which suggested that the content of HHE could be used to estimate the oxidative deterioration of dry‐cured fish products.

## Graphical abstract

In this study, dry‐cured Spanish mackerel (Scomberomorus niphonius, DCSM) was prepared via three different methods (hot‐air drying, cold‐air drying, and sun drying). The content of 4‐hydroxy‐2‐hexenal (HHE) and 4‐hydroxy‐2‐nonenal (HNE) derived from lipid oxidation in whole processes was investigated by HPLC‐MS/MS. The changes in fatty acid composition were detected by GC‐MS, and the degree of lipid oxidation were evaluated by the levels of acid values (AV), peroxide values (POV), thiobarbituric acid‐reactive substances (TBARS).

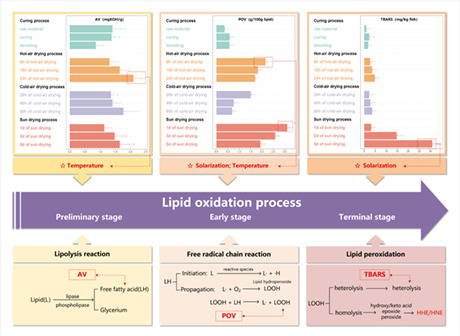

## 1 INTRODUCTION

4‐Hydroxy‐2‐alkenals, including 4‐hydroxy‐2‐nonenal (HNE) and 4‐hydroxy‐2‐hexenal (HHE), received considerable scholarly attention in the last few decades (Papastergiadis et al., 2014). These compounds are extraordinarily reactive due to their aldehyde groups and unsaturated moiety. Studies over the past two decades have provided important information about the production mechanism of exogenous HHE and HNE: The production of HNE is due to the attack of hydroxyl radicals on the n‐6 PUFA, especially arachidonic acid (ARA) and linoleic acid (Sakai et al., 1995; Tamura & Shibamoto, 1991), while peroxidation of n‐3 PUFA releases 4‐HHE (Soulage et al., 2018). The C=C bond and the C=O group in HHE/HNE formed a conjugated system in which the mobile π‐electrons provide a partial positive charge to C3. The negatively charged carbonyl oxygen atom promotes the withdrawal of mobile electron density from the β‐C, resulting in a regional electron deficiency status, so HHE/HNE is considered a soft electrophilic (Schaur et al., 2015). These electrophilic aldehydes react rapidly with nucleophilic sulfhydryl groups, which trigger carbonyl stress and involve in protein and DNA modification, consequently leading to structural damage and interfering with many pathophysiologic processes (Soulage et al., 2020). They can negatively impact various diseases, including Alzheimer's disease, atherogenesis, diabetes, and cancer (Bradley et al., 2010; Jakovčević et al., 2020). Recent studies have also observed that HNE is strongly associated with mortality outcomes from aggressive COVID‐19 (Žarković et al., 2021). Endogenous HHE/HNE is formed when the antioxidant capacities of cells and tissues are out of balance with reactive oxygen species (Jakovčević et al., 2020). However, exogenous HHE/HNE from the diet might also be a potential health risk. To date, no large‐scale studies have investigated the toxicological data of exogenous HHE/HNE. Only a few research teams have established reference thresholds by applying the threshold of toxicological concern (TTC) principle. A TTC level of exposure of 1.5 μg (kg body weight day)^−1^ was accepted for HNE and HHE (average body weight = 60 kg). These researchers found that regular consumption of cured meat products could be a potential risk (Papastergiadis et al., 2014; Surh & Kwon, 2005). Therefore, this indicates a need to evaluate the exposure of HHE and HNE in cured fish products. Spanish mackerel (*Scomberomorus niphonius*) has been one of the most popular marine fishes in coastal areas of China for a long history for its delicious taste and high nutritional value (Chang et al., 2021). It is also an important economic fish species in China, given its fast growth and high‐yield characteristics (Wan et al., 2020). However, it is difficult to maintain good quality for postmortem Spanish mackerel since, like most marine fishes, fresh Spanish mackerel is highly susceptible to spoilage and deterioration due to its biological composition. The abundant high‐quality protein (Hwang et al., 2019) and polyunsaturated fatty acids (PUFA) (Sun et al., 2020) degrade into easily digestible nutrients for microbial growth (Zheng et al., 2020). To date, there is a wide range of techniques for fish preservation, mainly in a single or combined form of freezing, salting, drying, and smoking (Baten et al., 2020), and of which, drying is one of the old and traditional methods of fish preservation in China. Dry‐curing combines salting and drying, extending the storage time and improving the textural profile and sensory quality of fish. Dry‐cured Spanish mackerel (DCSM) is a common and traditional preserved fish product. A large part of the harvested Spanish mackerel was made into DCSM annually. Despite its popularity among Chinese consumers, little attention has been paid to the potential hazards generated during its preserving production, such as lipid peroxidation (LPO)‐derived contaminants during dry‐curing. Spanish mackerel is rich in lipids, containing about 9.7%. During the dry‐curing process, LPO would happen and lead to the generation of a cascade of intermediate and end products (Sakai et al., 2000), including HHE and HNE. In fish meat, the amount of n‐3 PUFA is much higher than that of n‐6 PUFA, so the content of HHE may be relatively higher in fish, which leads to a more significant impact on edible safety (Sakai et al., 2014). Accordingly, the necessity of examination of the concentrations of HHE and HNE in DCSM is emphasized nowadays. This study aimed to investigate the relationship between HHE/HNE and PUFA in DCSM under different processes and speculate on their formation mechanisms. Specifically, the following issues will be addressed: (a) exposure assessment of HHE and HNE in DCSM was conducted to analyze which processing factors influenced the LPO; (b) analyze the correlation between PUFA with HHE, HNE, and TBARS, then further investigate the possible mechanism of HHE/HNE occurrence in DCSM during different processes; and (c) evaluate the applicability of HHE/HNE as new indicators of LPO in processed fish products by establishing models. Hopefully, this research will contribute to a deeper understanding of typical chemical hazards in DCSM.

## 2 MATERIALS AND METHODS

### 2.1 Materials and chemicals

Fresh raw Spanish mackerel was purchased from a local market in Qingdao, China. After beheading and eviscerating, fish samples were cut into uniform steaks having about 200 g weight. Sodium chloride of food grade was purchased from China National Salt Industry Corporation. NaOH, 2,4‐dinitrophenylhydrazine (DNPH, 97%), hexane, chloroform, and butylated hydroxytoluene (BHT) was purchased from Sinopharm Chemical Reagent, Ltd (Shanghai, China). Methanol (chromatographically pure) (Oceanpak, Sweden); (2 E)‐4‐hydroxy‐2‐hexenal (HHE, ≥99%), trans‐4‐hydroxy‐2‐nonenal (HNE, ≥99%), (2 E)‐4‐hydroxy‐2‐hexenal‐d3 (HHE‐d3, ≥99%), and trans‐4‐hydroxy‐2‐nonenal‐d3 (HNE‐d3, ≥99%) standards were provided by Alta Technology Co., Ltd. All the standard solutions were kept at −80°C.

## 3 SAMPLE PREPARATION

**FIGURE 1:**
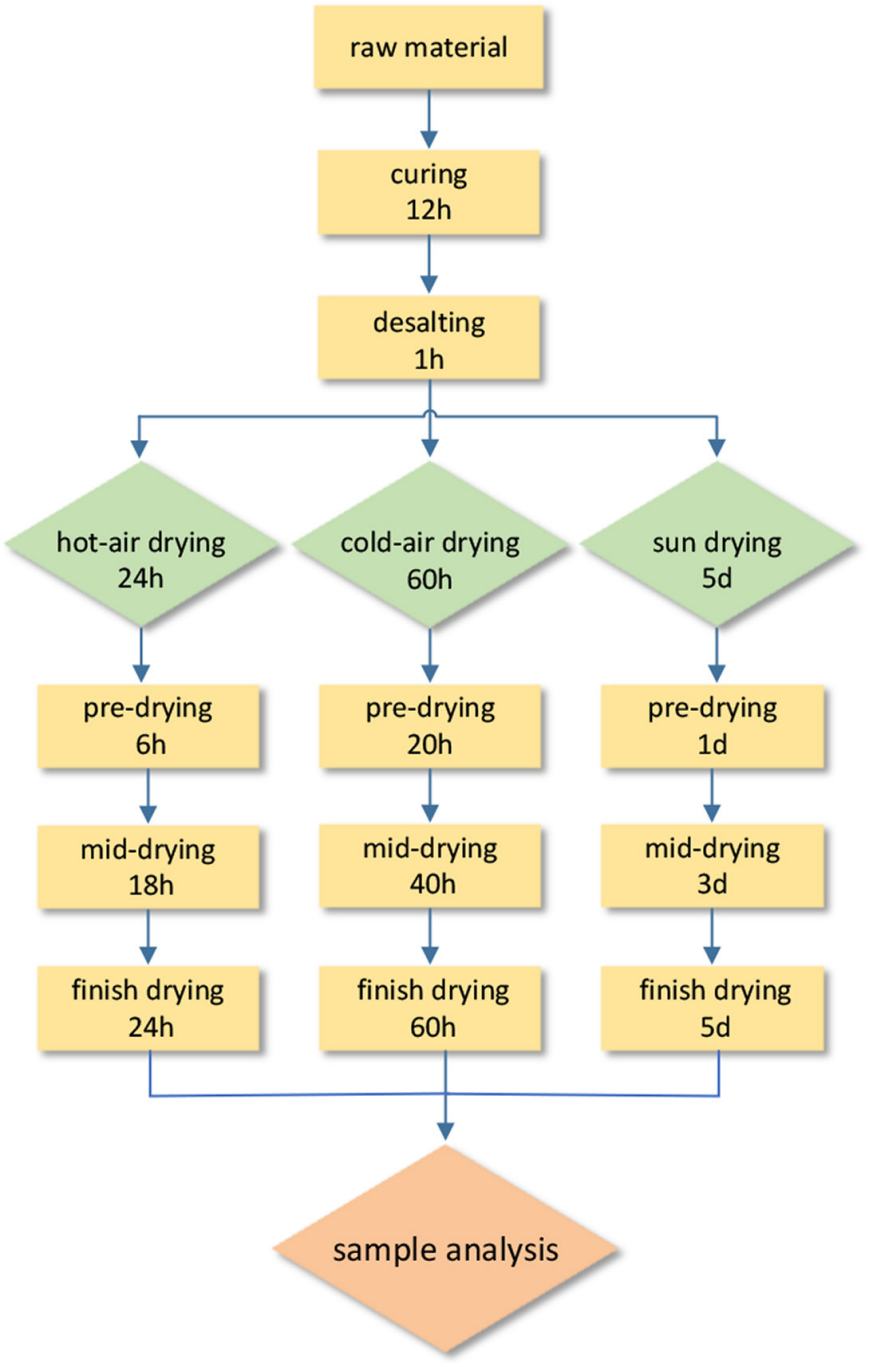
The whole stage of processing. Yellow rectangles represent the sampling points during processing.

The pretreated Spanish mackerel steaks were marinated in 10% sodium chloride solution (w/v) at 4°C for 12 h. The cured fish were carefully washed with distilled water to remove the remaining salt before drying. After that, the samples were divided into three parts and separately dried by hot‐air‐drying, cold‐air‐drying, and sun‐drying methods until the water content of cured fish decreased to about 30%. The hot‐air‐drying group was kept at 60°C for 24 h in a drying oven (Zhongshan Yinfu Electric Appliance Co., LTD). The cold‐air‐drying group was kept at 10°C for 60 h in an artificial climate control box (Yixi Instrument Equipment Co., LTD). The sun‐drying group was dried by exposure to ambient sunlight for 5 days. The average wind velocity was 12 km h^−1^, and the temperature was recorded (5–12°C). During sun drying, the fish were placed in hanging nets to ensure homogenous drying and to prevent contamination by insects and other pests. After drying, all samples were packaged with transparent polyethylene bags and stored at −20°C until use. Samples were thawed at 4°C for 24 h before any analysis. Figure 1 shows the sampling points during processing.

## 4 ANALYSIS OF **HHE** AND **HNE** IN **DCSM**

The analysis of HHE and HNE in DCSM was performed according to the method of Douny et al. (2015). Two grams of the crushed sample containing 50 μl internal standard solution (10 mg L^−1^) was added to 15.0 ml derivative solution (0.5 mg ml^−1^ DNPH in 1 N HCl solution) with 25 mg of BHT. The mixture was derivatized for 2 h in a dark environment. Then, 30 ml dichloromethane was added to the mixture, and the supernatant was removed after centrifuging (7000 r/min) and repeated twice. The extracting solution volume was fixed to 100 ml, then isolated and purified with SPE, concentrated, and analyzed by LC–MS/MS (6460, Agilent) with an electrospray ionization (ESI) system equipped with Agilent MassHunter Quantitative Analysis. An ACQUITY UPLC HSS T3 column (100 mm*2.1 mm*1.8 μm) (Agilent Technologies Co., Ltd.) was selected for analysis. The solvent flow was 0.3 ml min^−1^, and the column temperature was set at 40°C. The injection volume was 5 μl. The mobile phase was acetonitrile and acetic acid solution at pH = 3.55. The gradient elution conditions were from 40% to 65% of acetonitrile in 10.0 min and from 65 to 100% in 4.0 min; then, the contribution of acetonitrile was decreased to 40% over 1.0 min, and maintained for another 3.0 min of reconditioning. The ESI‐MS analysis was performed in negative ionization for all compounds. The capillary temperature was set at 300°C, and the spray voltage was 70 kV. HHE‐d3 and HNE‐d3 were used as the internal standard for quantification, and the results were expressed as μg kg^−1^ fish.

## 5 FATTY ACID COMPOSITION ANALYSIS

The fatty acid composition was analyzed using the method of Ma et al. (2020) with slight modifications. Briefly, 10 mg of lipid in the samples was extracted by hydrolysis–ether solution. Eight milliliter methanol of 2% NaOH was added to the lipid samples, and the mixture was refluxed at 80°C ± 1°C until the oil drops disappeared. Then, 7 ml of 15% BF3‐methanol solution (w/w) was added. Fatty acid methyl esters (FAMEs) were extracted by N‐heptane and filtered through a 0.22 μm nylon needle filter. The fatty acid profiles were determined using a capillary column gas chromatograph–mass spectrometer (GC–MS, 7980A/5975C, Agilent). FAMEs were separated using a polydicyanopropylsiloxane strong polar stationary phase (100 m*0.25 mm*0.2 μm) and N2 as a carrier gas. The injector temperature was 270°C, and the injection volume was 1 μl with the spit ratio of 100:1. The programmed temperature was set as follows: the initial temperature was 100°C for 13 min; increased to 180°C at 10°C/min and held for 6 min; then increased to 200°C at 1°C/min and held for 20 min; and finally, increased to 230°C at 4°C/min and held for 10.5 min. The retention time of samples was compared with the standard FAME mixtures for qualitative analysis, and the area normalization method was used for quantitative analysis.

## 6 MEASUREMENT OF ACID AND PEROXIDE VALUES

Official methods of the Ca 5a‐40 of the American Oil Chemists Society (AOCS) were used to determine the acid values (AV) and peroxide values (POV) (Cd 8b‐90) of each sample after extracting the total lipids.

## 7 MEASUREMENT OF ***TBARS***

The analysis of TBARS in DCSM was performed according to the method of Papastergiadis et al. (Papastergiadis et al., 2012). Approximately 5 g of sample was derivatized with TBA reagent. Ten microliter of the sample was injected into a ZORBAX Extend‐C18 HPLC column (5 μm, 150 × 4.6 mm) (Agilent Technologies Co., Ltd.) and held at 30°C. The mobile phase consisted of 10 mM CH₃COONH₄, and methanol (70:30, v/v) was pumped at 1.0 ml min^−1^. The detection wavelength of the UV detector was set at 532 nm. The standard curve was plotted by taking the concentration of the standard solution and sample to be tested in the high‐performance liquid chromatography (1260 Agilent), and the corresponding peak areas were measured. The concentration of MDA in the solution to be measured was obtained from the standard curve.

## 8 STATISTICAL ANALYSIS

Statistical analyses were performed using Origin 2021 software (OriginLab Corporation). Significant differences were identified using Duncan's multiple‐range tests (95% confidence level, p‐value <0.05). Data management and analysis were performed using SPSS 26.0 (IBM, SPSS Inc). Heatmaps and hierarchical cluster analysis were performed using R 4.2.1.

## 9 RESULT & DISCUSSION

### 9.1 Evolution of ***HHE*** and ***HNE*** in ***DCSM*** during processing

**FIGURE 2:**
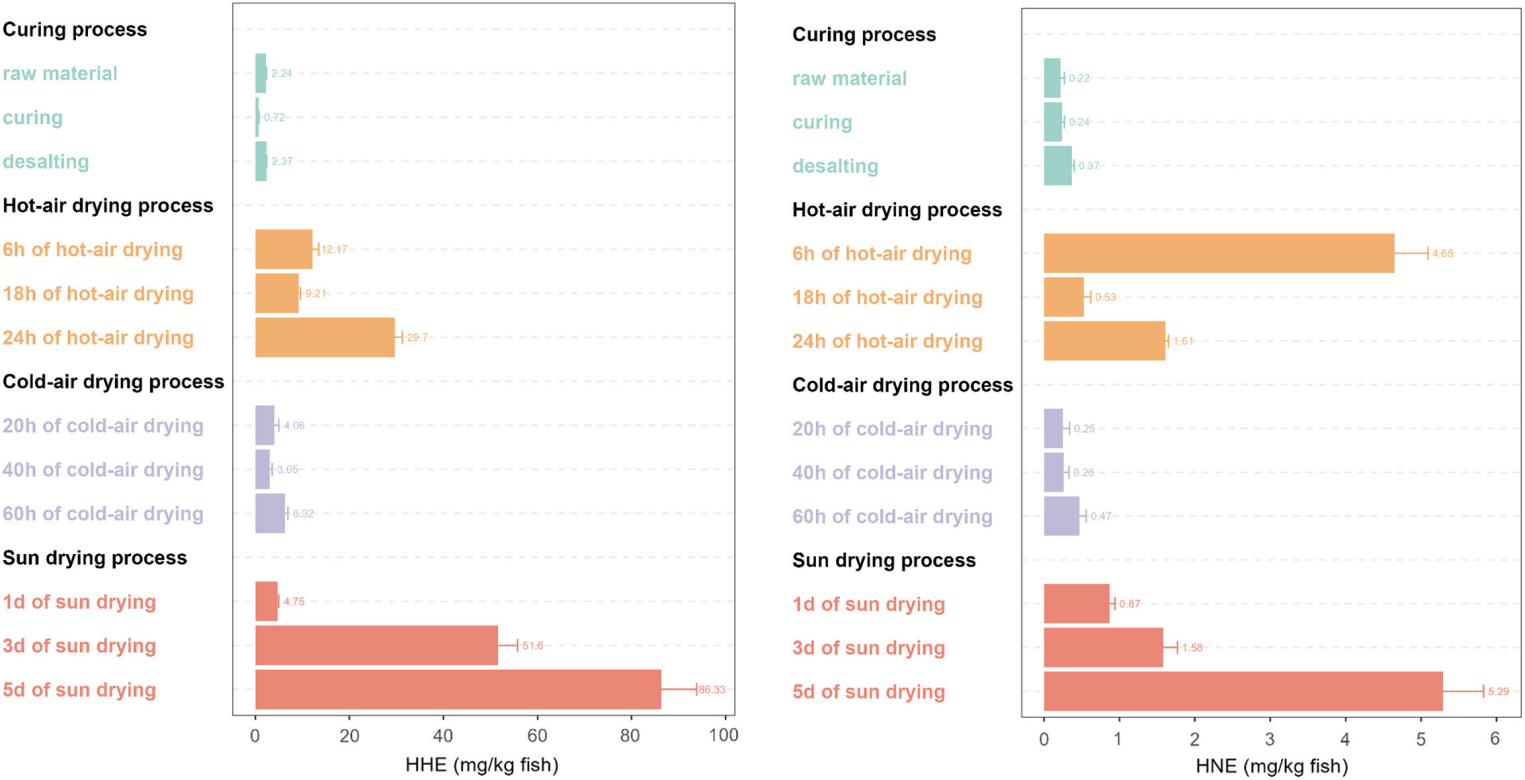
Evolution of the HHE and HNE contents in DCSM in different types of each processing period. Data are means ± SD of three independent experiments.

Figure S2 displays the chromatograms of HHE and HNE, and the contents of HHE and HNE at each sampling point during processing are shown in Figure 2. In this study, the whole dry‐cured process was divided into two processing. The first was the curing and desalting processing, in which sodium chloride was the main factor affecting the content of HHE/HNE. The second was the drying process; the temperature (cold‐/hot‐air drying) and solarization (sun‐drying) affected the occurrence of HHE/HNE. Starting from the curing and desalting processing: the content of HHE decreased significantly from 2.24 ± 0.92 to 0.72 ± 0.38 mg/kg fish after curing but increased to 2.37 ± 1.03 mg/kg fish after desalting. These results reflected those of Munasinghe et al. (2006), who also found that HHE formation at 0°C storage was suppressed by sodium chloride. The exact formation mechanism of HHE in fish muscles was very complicated (Sakai et al., 2008). A possible explanation for this might be that sodium chloride accelerated the addition reaction of HHE and protein. Meanwhile, the content of HNE kept increasing from 0.22 ± 0.11 to 0.37 ± 0.14 mg/kg fish after curing. These results were in good agreement with the previous studies, such as Surh and Kwon (2005), who found that the content of HNE in salted mackerel was 0.02 mg/kg fish higher than raw mackerel (0.01 mg/kg fish); Sakai et al. (2004) also showed a certain content of sodium chloride could accelerate the formation of HNE in minced pork. A possible explanation for this might be that the rapid decomposition of n‐6 PUFA was accelerated by the addition of sodium chloride. Although these results differ from some published studies (Sakai, Ohtsubo, et al., 2006; Sakai, Shimizu, & Kawahara, 2006), they found sodium chloride had negative impacts on the occurrence of HNE in boiled pork. This discrepancy could be attributed to the different cooking methods, some cooking methods, such as drying, boiling, and frying, may affect the formation and disappearance mechanism of HNE in samples (Weber et al., 2008). Therefore, it is possible that the accelerating effect of sodium chloride on HNE formation may only be present in samples without other processing. Turning to the experimental evidence on the fluctuation of HHE/HNE content during the drying processing, it was found that HHE and HNE decreased or remained constant at first in both hot‐air‐ and cold‐air drying stages, then increased significantly in the end stage of drying. The content of HHE/HNE at the end stage in cold‐air drying (6.32 ± 0.92 mg/kg fish) was lower than in hot‐air drying (29.69 ± 5.52 mg/kg fish). These results seemed consistent with other research, which found that compared with freeze‐drying, the high concentration of linoleic acid and heme in foodstuffs had a more significant effect on the occurrence of HNE (Gasc et al., 2007). There were several possible explanations for these results. Firstly, Zhang et al. (2015) supposed that these low‐molecular‐weight aldehydes were easily volatilized at high temperatures. Secondly, in the present study, Liu et al. (2020) also observed that the concentrations of HHE and HNE decreased at the beginning of extreme frying temperatures, and they proposed that high temperatures cause lipid exchange and aldehyde migration between samples and oils. This finding was also reported by Meinert et al. (2007), who also reported that extreme frying temperatures resulted in a reduction in low‐molecular‐weight aldehydes in pork fat. They suggested that these aldehydic by‐products might escape into a certain medium. Thirdly, these aldehydes may react under certain conditions with food components such as protein and amine‐containing phospholipids (Weber et al., 2007). Together these results provided essential insights into HHE and HNE sensitive to temperature, which caused complicated variations when the temperature was too high or too low. During cold‐air drying, the low temperature influenced the formation of HHE/HNE as the lipase activity was impacted by the lower temperature. Only a small amount of free fatty acids was oxidized, which affected the accumulation of lipid hydroperoxides (LOOH•) and slowed down the subsequent LPO process (Wang et al., 2018). However, during the sun‐drying progress, the content of HHE continuously increased from 4.75 ± 2.22 mg/kg fish to 86.33 ± 10.54 mg/kg fish, and the content of HNE also kept rising about 4.42 mg/kg fish. There were two likely causes for this observation. On the one hand, the occurrence of alkenals was time and temperature dependent in most species (Siliang et al., 2021). Due to the time of sun‐drying process being the longest, the contents of HHE and HNE increased approximately 4‐ ~ 5‐folds compared with DCSM in hot‐air drying. On the other hand, solarization, heating, and metal ions such as copper and iron, and heme pigments such as myoglobin and hemoglobin, could promote the auto‐oxidation of lipids (Sakai, Ohtsubo, et al., 2006). As mentioned above, these factors contributed to the deepest lipid oxidation in sun‐drying process. Indeed, the n‐3 PUFA contents (Table S1) displayed the same tendency as the HHE/HNE content, with the greatest decrease (3%) during sun‐drying. Together these results provide important insights that the n‐3 PUFA content has a more substantial impact on the formation of unsaturated aldehydes in the fish medium than n‐6 PUFA. Overall, these figures were quite revealing in several ways. Firstly, different drying methods significantly affected the formation of HHE and HNE, and DCSM under sun drying in the traditional Chinese style had higher contents of these aldehydes. Secondly, the fluctuation of HHE and HNE contents in hot‐drying process accorded with previous research, which showed the migration of aldehydes in lipids at high temperatures. Finally, as mentioned earlier, the content of HHE was higher by 18.44‐, 13.45‐, and 16.32‐folds compared with that of HNE after three different processes, respectively. The results in this chapter indicate that the formation and accumulation of HHE/HNE in fish are very complicated during processing. The next chapter, therefore, discussed the relationship between lipid oxidation and PUFA.

## 10 CHANGES IN FATTY ACID COMPOSITIONS AND OTHER OXIDATION INDICES

**FIGURE 3:**
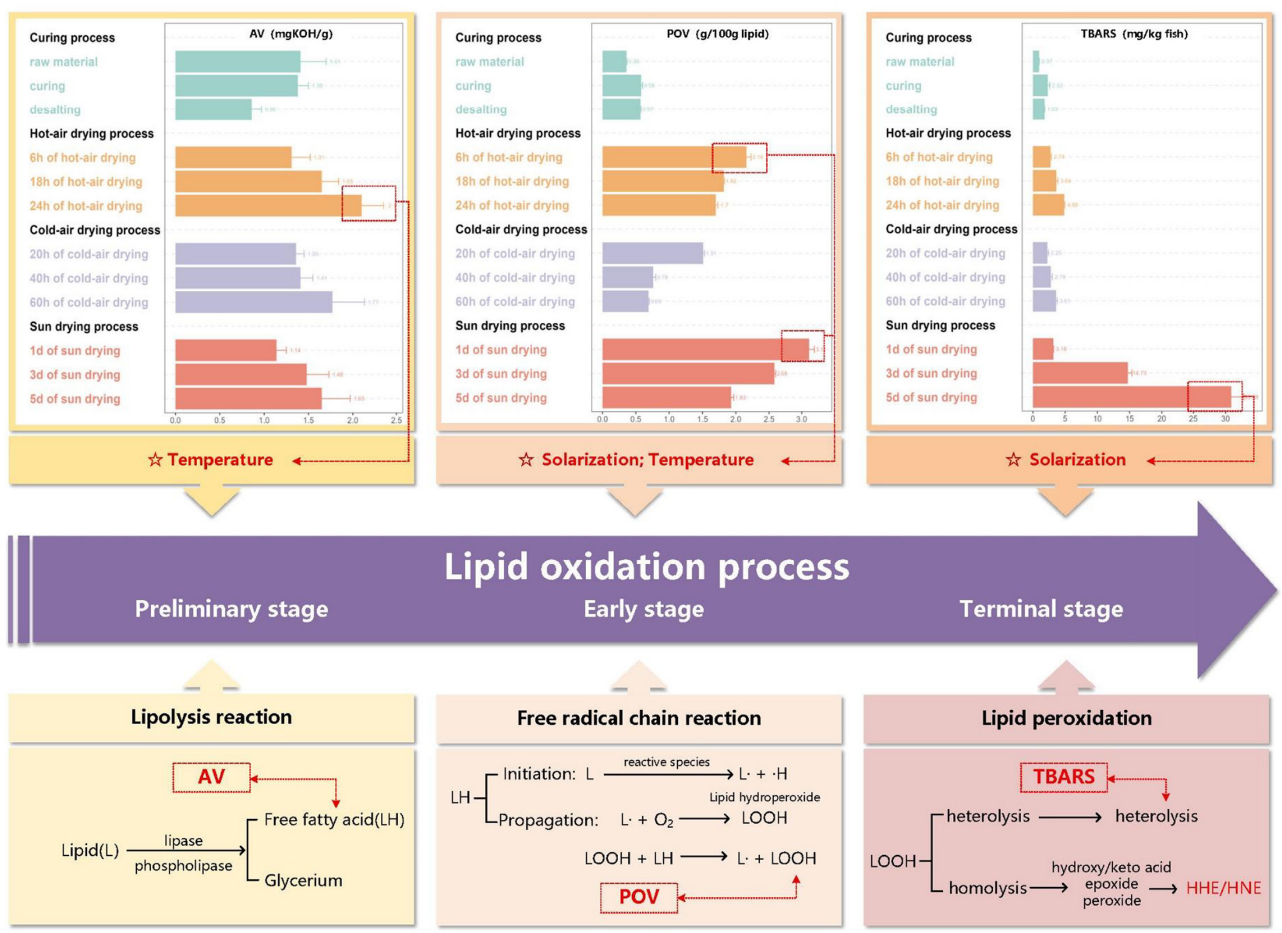
Evolution of the lipid oxidation index in DCSM and effect of processing factors on lipid oxidation processes.

Changes in fatty acid composition were monitored at each stage in three drying processing. Figure S3 displayed the fatty acid GC spectrums. As shown in Table S1, the difference between SFA and PUFA indicated that fatty acid oxidative decomposition coincided with low‐molecular‐weight aldehydes formation and accumulation. Notably, Table S1 illustrated the large proportion of PUFA (28.90 ± 0.65%) in raw Spanish mackerel, especially n‐3 PUFA (26.60 ± 1.03%) and n‐9 PUFA (31.30 ± 0.71%), while the content of n‐6 PUFA was very little in DCSM (<2.30%). This proportion of fatty acids mirrored those of the previous studies which show that raw salmon contains 27.6 ± 0.3% of PUFA and 1.6% of n‐6 PUFA (Larsson et al., 2016). An implication of this was the possibility of a more considerable amount of HHE. Moreover, different drying processing significantly changed the fatty acid content; what stood out in this table was the rapid decrease in the contents of PUFA and n‐3 PUFA after the drying process. For instance, the contents of PUFA and n‐3 PUFA were 24.8 ± 0.80% and 22.5 ± 1.07% at the end stage of sun‐drying processing. In the hot‐drying processing, PUFA and n‐3 PUFA content remained constant and then decreased with corresponding values of 27.4 ± 0.62% and 25.1 ± 1.21% at the end stage. Conversely, no statistically significant reduction in the content of n‐3 PUFA was found in cold‐air‐drying processing. Taken together, these results seemed to be consistent with other research which found the occurrence of 4‐hydroxy‐2‐alkenals appeared to be governed by PUFA originally present in foods (Surh et al., 2007). Changes in lipid oxidation indices including AV, POV, and TBARS were also examined during the whole processing. Figure 3 below illustrated that lipid oxidation was an ever‐changing process in which the changes in AV, POV, and TBARS showed the degree of oxidation at different stages. AV represented free fatty acids produced by specific lipases and phospholipases that decompose fatty acids from the phosphor glycerol framework (Song et al., 2017). There was a clear trend of decreasing after desalting and increasing significantly during all drying processes in AV. The highest AV was observed at the end stage in the hot‐air‐drying process (2.10 ± 0.15 mg KOH g^−1^). This result is in agreement with Ma, Liu, and Liu (2019) findings which also showed the consistent rising of AV during 30 days of heating in a 60°C oven. The hypothesis could partly explain these observations that high temperature increased lipase activity inside the fish muscle and accelerated LPO. POV was widely used to evaluate the degree of lipid chain reaction, which indicated the levels of LOOH•. POV was increased after curing, then reached the highest point in the early stage of the drying process with corresponding values of 2.16 ± 0.07, 1.51 ± 0.02, and 3.11 ± 0.08 g/100 g lipid (Table S1), respectively. The increase in POV at the predrying stage in this study corroborated those earlier findings: in the initial oxidation step, oxidation of monoenoic and polyenoic acids resulted in the formation of different LOOH• (Xu et al., 2022). However, POV kept decreasing in the subsequent drying process. The observed decrease in POV in the later drying process could be attributed to the rapid decomposition of hydroperoxides, as they were unstable in high temperatures (Hu et al., 2022). TBARS mainly represented the various substances produced at the end stage of lipid oxidation. There was a similar trend between the content of TBARS and AV during the drying process, while in the curing and desalting processing, the content of TBARS rose after curing but declined after desalting. The content of TBARS increased to a peak at 30.88 ± 1.91 mg/kg fish at the end stage of sun drying, ranking the first among all groups. Soulage et al. (2020) also found that the accumulated TBARS content of the sun‐drying group was much higher than that of other groups. These findings may be explained by the fact that the degree of LPO was deepened by heating or solarization. As mentioned earlier, the oxidation rate was significantly increased when exposed to solarization, myoglobin, and other mediated photooxidation reactions were activated. A large amount of LOOH• was further decomposed or hot split (Schaur et al., 2015). According to these data, as seen in Figure 3 above, the higher temperature could be a major factor that accelerated the initial rate of lipid oxidation. Exposure to higher temperatures and solarization promoted the process of lipid chain reaction. Compared with higher temperatures, exposure to the sun had a more significant impact on the lipid chain reaction and the LPO stage.

## 11 RELATIONSHIP BETWEEN ***HHE*** / ***HNE*** OCCURRENCE AND OXIDATION INDICES

**FIGURE 4:**
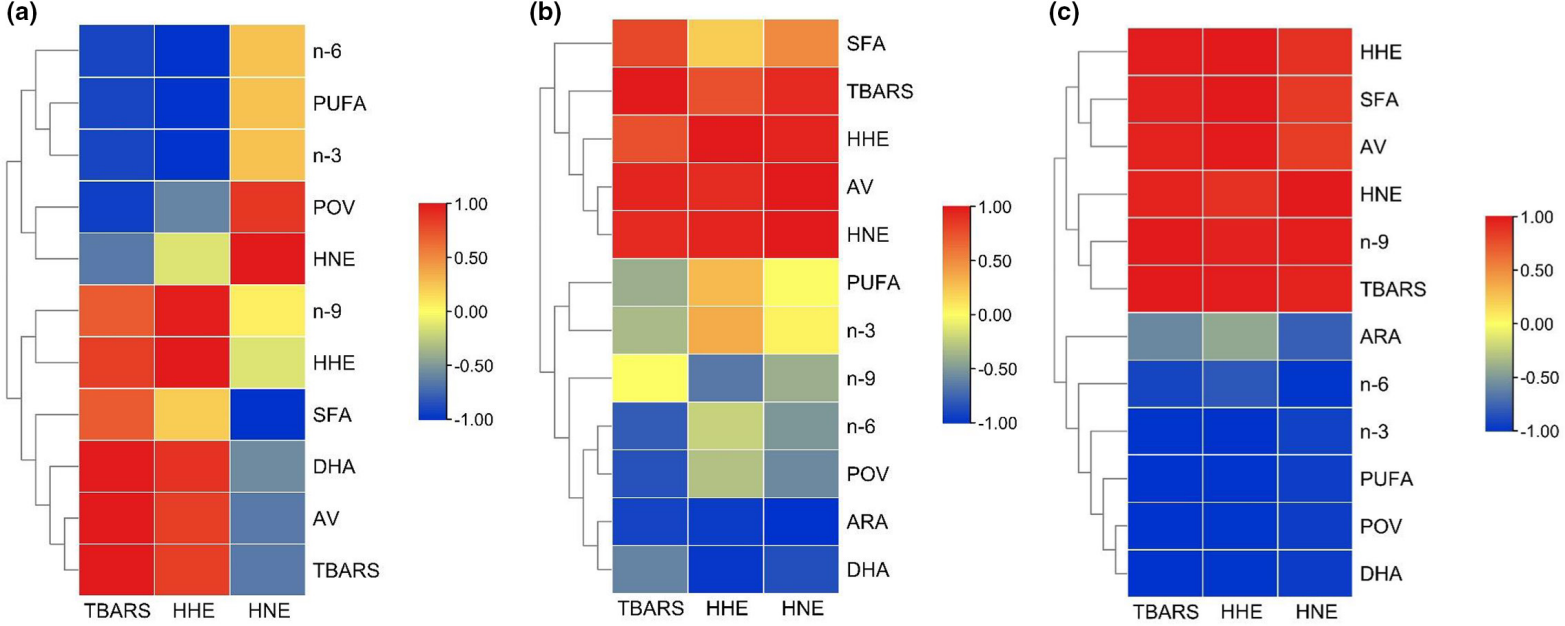
Pearson correlation heatmap and dendrogram of the different indexes by hierarchical cluster analysis of tested fishes in different processing periods.

A Pearson correlation analysis was applied to assess the relationship between HHE/HNE and fatty acid composition in DCSM under three processing conditions. This chapter's purpose was to investigate these unsaturated aldehydes formation mechanisms further. Statistically significant levels of the correlational analysis are displayed in Table S2 The hierarchical clustering heatmaps were drawn using the calculated Pearson correlation coefficient to indicate the correlation between the HHE/HNE (y‐axis) and the other oxidation indicators (x‐axis) (Figure 4). In addition, the correlations between TBARS, commonly used as a biomarker for lipid peroxidation (Tullberg et al., 2019), and other oxidation indices were also calculated as a control group. Figure S1 displays the representative HPLC chromatograms of TBARS. As shown in Figure 4, an extremely significant correlation was found between TBARS and AV (*p* < .01) in hot‐drying conditions and had a strong correlation under sun‐drying (*p* > .05). These results suggested changes in the degree of LPO during drying processing. For HHE and HNE, a significant negative correlation was found between HHE formation and n‐3/6 PUFA (*p* < .05) content in hot‐air‐drying and sun‐drying processes (Figure 4). HHE also had small correlations with n‐6 PUFA content in the cold‐air‐drying process, which confirmed that n‐3/6 PUFA were the main precursors for HHE formation during LPO. Results from earlier studies demonstrated a strong and consistent association between HHE/HNE and n‐3/6 PUFA (Albuquerque et al., 2022), while the mechanism of occurrence of HHE and HNE had not been fully elucidated. Therefore, the possible pathways for HHE formation from n‐3/6 PUFA were deduced based on the previous work. As mentioned before, HNE was strongly influenced by heating, and no negative correlation was found with PUFA in hot‐air‐drying progress. A correlation between HNE and n‐6 PUFA content was only found in the sun‐drying progress, and the result is significant at the *p* = .05 level. A previous study has demonstrated that HNE only originated from n‐6 PUFA methyl esters, they also observed that n‐6 PUFA played a pro‐oxidant role (Ma, Liu, Cheng, & Liu, 2019). Our research also showed that the correlation between the HNE and ARA (n‐6 PUFA) content was significant in cold‐air‐ (*p* < .01) and sun‐drying processes. These results are consistent with previous observational studies, which proposed that ARA yields more HNE than other fatty acids (Alghazeer & Howell, 2008). Hence, the possible formation pathways were proposed to assess the feasibility of linoleic acid and ARA as the precursor of HNE. One surprising variable that was found to be strongly associated with HHE was n‐9 PUFA content (*p* > .05) in two conditions, and HNE also had a strong correlation with n‐9 PUFA content (*p* > .05) in the sun‐drying period. However, Ma et al. (2020) found HHE absent in n‐9/6‐type oils, and HNE was less in n‐9‐type oils. Hence, it could conceivably be hypothesized that the n‐9 PUFA played the role of promoting oxidation in HHE/HNE formation and accumulation but not as a precursor. The significant positive correlations of HNE (*p* < .05) with MUFA were interesting but unsurprising. These results are in agreement with Surh et al.'s (2010) findings which also showed positive correlations between MUFA and HNE (*r* = .297, *p* = .070) in the food lipids. A significant correlation (*p* < .05) between SFA and HHE/HNE was also observed. This result may be explained by the oxidation of PUFA, which also accords with the significant negative correlation (*p* < .05) between HHE/HNE and PUFA. It can, therefore, be assumed that the occurrence of HNE/HHE could be affected by the amount of n‐6/3 PUFA and the presence of MUFA and SFA (Surh et al., 2010). This combination of findings supported the conceptual premise that the oxidative interactions among fatty acids could accelerate the autoxidation of fatty acids that could produce 4‐hydroxy‐2‐alkenals.

## 12 ANALYSIS OF HHE/HNE POSSIBLE FORMATION PATHWAYS

**FIGURE 5:**
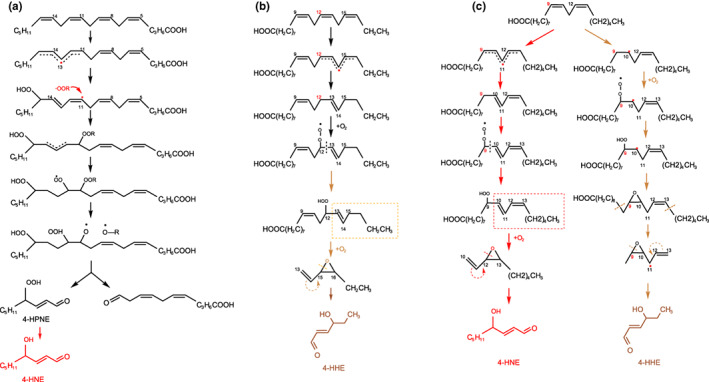
(a) Possible HNE formation pathways in ARA(n‐6). (b) Possible HHE formation pathway through the peroxidation of linolenic acid (n‐3). (c) Possible HHE and HNE formation pathway through the peroxidation of linoleic acid (n‐6).

LPO was a typical free radical chain reaction involving reactive oxygen species. As shown in Figure 5, this reaction contained the stages of initiation, propagation, and termination (Altomare et al., 2021). The initiation stage started with a strongly reactive starting radical, which was usually generated due to radiation/heat‐induced homolysis of covalent bonds. In PUFA, for example, the C=C weakened the C‐H between the carbon and hydrogen atoms attached to it, so the hydrogen atoms of the methylene (‐CH_2_‐) connected to the C=C units were easily abstracted, which generated a lipid radical (L•) after leaving a lone pair of electrons. After the molecular rearrangement of L•, the C=C would be conjugated. Conjugated dienes combined with molecular oxygen under aerobic conditions to form a lipid peroxyl radical (LOO•), which could extract hydrogen from another adjacent lipid molecule to produce a LOOH and a second L•. This way, the LPO chain reaction formed a cycle, forming a large amount of LOOH. A note of caution is due here since LOOH could be produced by both autoxidation and photooxidation in nonenzymatic oxidation (Zhou et al., 2020), and the autoxidation of linoleic acid is an example in this study. LOO• also could form cycloperoxides and intryclic peroxyradicals through intramolecular C=C addition, which formed various short‐chains‐reactive carbonyl species. As mentioned above, more free radicals would be generated when UV or heat sources existed, and the LPO chain reaction would be prolonged. It resulted in the continuous consumption of lipid molecules and generation of LOOH and LOO•. Thus, the content of HHE/HNE in DCSM under hot‐air‐ and sun‐drying conditions was higher and significantly negatively correlated with PUFA. Based on the LPO chain reaction, the pathways for HHE and HNE formation via PUFA oxidation could be proposed. As presented in Figure 5a–c, HNE most likely originated from the breakdown of the ARA, linoleic acid, or their hydroperoxides (Uchida et al., 1999). ARA was susceptible to free radical reaction to form 15‐hydroperoxyeicosatetraenoicacid (15‐HpETE), and the addition of peroxy radicals into the C‐11,12 of 15‐HpETE could form a peroxide by oxidization and abstraction of the hydrogen atom, which then led to the formation of 4‐hydroperoxy‐2 E‐nonenal (HPNE) after the rupture of peroxide bonds, and the final peroxidase was converted HNPE into HNE (Zhou et al., 2020). Linoleic acid was a dienoic acid, the position where the formed conjugate was on the C‐11 atoms between the two C=C units, so HNE was derived from the breakdown of the 9‐LOOH of linoleic acid. HHE was most likely yielded by the decomposition of 12‐LOOH of linolenic acid, which broke down to form intermediates with C‐6 atoms. The correlation between HHE and n‐6 PUFA was strong, this also accords with the observation by Ma et al. (2020), which showed that high concentrations of n‐6 PUFA could produce HHE. C=C could be directly oxidized while forming 12‐LOOH and C‐6 atoms intermediate with an epoxy structure.

## 13 APPLICABILITY OF HHE/ ***HNE*** AS OXIDATION INDICATORS OF DRY‐CURED FISH PRODUCTS

**FIGURE 6:**
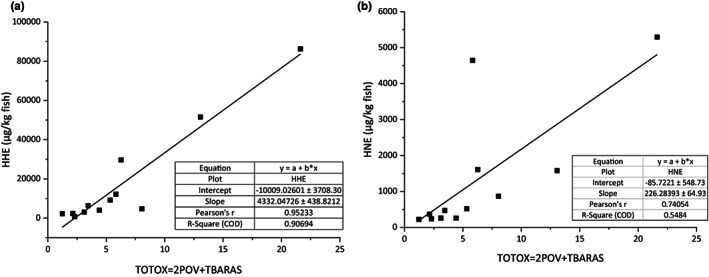
Linear correlations of FFFHHE and HNE with TOTOX during the whole stage of processing.

In this study, the aim was to assess the applicability of HHE/HNE as characteristic aldehydes in dry‐cured fish products investigated. Prior studies have noted the possibility that HNE could be used as an indicator of oxidation, which reflected the change in fish's textural quality and nutritional value (Alghazeer & Howell, 2008). Traditionally, the total oxidation (TOTOX) value has been assessed by measuring the POV and *p*‐anisidine value. Since the *p*‐anisidine value was mainly used in oil samples, TBARS also measured the extent of LPO products. Based on the above data, the TOTOX value was calculated based on POV and TBARS, using the formula (TOTOXTBA = 2 POV + TBARS) as described by Hu et al. (2022). Wanasundara and Shahidi (1995) also considered the TOTOX_TBA_ value as an essential indicator for indicating the degree of oxidative deterioration of lipids. A regression analysis was conducted between the HHE/HNE content (y) and TOTOX value (x) during the whole stage of processing. It can be seen from the data in Figure 6a that HHE had a strong correlation with TOTOX in DCSM (Pearson's *r* = .952), and HNE also had a good correlation with TOTOX. Although the Pearson correlation coefficient between HNE and TOTOX was 0.740, the fitted line displayed a low *R* ^2^ value (.548). Comparing the results with other research results, it is confirmed that the applicability of HNE as a food quality marker only existed in those foodstuffs containing a high amount of n‐6 fatty acids (Sakai et al., 1995). These relationships may be explained by the lower n‐6 PUFA and HNE contents in fish products, and HNE were more sensitive to temperature, which could cause complicated variations in different processing. HHE and TOTOX in DCSM showed good linear relationships (*R* ^2^ value = .907), indicating that HHE was a good indicator for evaluating the LPO degree of dry‐cured fish products. These results also accorded with earlier observation (Ma et al., 2020), which showed that the content of HHE could evaluate the oxidation of n‐3‐type oil during storage. Based on the good linear trend between the content of HHE and TOTOX value in DCSM, this study proposed that HHE could be used as a new marker to evaluate the degree of LPO in dry‐cured fish products containing high levels of n‐3 PUFA.

## 14 CONCLUSION

In conclusion, the present study was designed to investigate the occurrence of HHE and HNE in DCSM. During different drying processing, considerable amounts of HHE and HNE were detected in samples. The heating promoted the initial stage of lipid oxidation, and exposure to the sun had more significant impacts than the heating on the following chain reaction stage and the LPO stage. The HHE/HNE contents were closely related to the fatty acid compositions. Due to the high content of n‐3 PUFA (26.60%) and very little content of n‐6 PUFA (2.30%) in Spanish mackerel, the content of HHE, which was mainly derived from n‐3 PUFA was 18.44‐, 13.45‐, and 16.32‐folds higher than that of HNE after three different processing, respectively. Considering higher levels of HHE in DCSM, the HHE could be classified as characteristic aldehydes in dry‐cured fish products. Based on the regression analysis, HHE showed a strong correlation (Pearson's *r* = .952) and a good linear relationship (*R* ^2^ value = .907) with the TOTOXTBA values, which suggested HHE could be used as a new indicator for evaluating the oxidation, especially the degree of LPO in dry‐cured fish products. The present work provided important information for understanding the HHE/HNE formation and alternative new oxidation indicators in dry‐cured fish products. It is important to note that dry‐cured fish products could be one of the exogenous sources of HHE/HNE. A further study could characterize different antioxidants and carbonyl trapping substances and assess the long‐term effects of reduction in HHE and HNE levels in fish products.

## FUNDING INFORMATION

This work was supported by the National Key R&D Program of China [2019YFD0901703].

## CONFLICT OF INTEREST

No potential conflict of interest was reported by the author(s).

## ETHICAL APPROVAL

This study does not involve any human or animal testing. ORCID *Cui Kexin* https://orcid.org/0000‐0002‐7049‐2843

## Supporting information


Appendix S1

## Data Availability

The datasets generated and/or analyzed during the current study are available from the corresponding author on reasonable request.
