# Supplementary material for: Effect of drying processes on the occurrence of lipid oxidation‐derived 4‐hydroxy‐2‐hexenal and 4‐hydroxy‐2‐nonenal in Spanish mackerel (Scomberomorus niphonius)
Source: Food Sci Nutr. 2022 Nov 15;11(2):1013–23. doi: 10.1002/fsn3.3138 (PMC9922132; doi:10.1002/fsn3.3138)
Supplement: Supplementary file 1 — Appendix S1 [file FSN3-11-1013-s001.docx]

**Supplementary data**

**Effect of drying processes on the occurrence of lipid oxidation derived 4-hydroxy-2-hexenal and 4-hydroxy-2-nonenal in Spanish mackerel (*Scomberomorus niphonius*)**

Kexin Cui^a,b^, Nan Liu^a^*, Yong Sun^a^, Guohui Sun^a^, Shanshan Wang^a^, Min Yang^a^, Xiaoli Wang^a,b^, Deqing Zhou^a^ , Yinggang Ge^a^, Dajun Wang^C^, Mingli Wang^d^

*^a^ Chinese Academy of Fishery Sciences Yellow Sea Fisheries Research Institute, Qingdao, China;*

*^b^College of Food Science and Engineering, Ocean University of China, Qingdao, China ;*

*^c^Yantai Haiyu Food Co., Ltd., Yantai, China;*

*^d^Penglai Huiyang Food Co., Ltd., Yantai, China*

*N. Liu, Mailing address: No.106 Nanjing Road, Shinan District, Qingdao 266071, China. Tel: (86)13953299840. E-mail: liunan@ysfri.ac.cn

**Table S1. Fatty acid composition (area %) and initial chemical properties of dry-cured Spanish mackerel (DCSM) in different processes** *^a^*

| parameter | Cured process | | | Hot air-drying process | | | Cold air-drying process | | | Natural drying process | | |
| --- | --- | --- | --- | --- | --- | --- | --- | --- | --- | --- | --- | --- |
|  | raw material | curing | desalting | early-stage | mid-stage | end-stage | early-stage | mid-stage | end-stage | early-stage | mid-stage | end-stage |
| C14:0 | 3.90±0.14d | 3.30±0.09f | 3.60±0.13e | 4.30±0.21c | 3.70±0.18e | 4.60±0.23b | 4.30±0.21c | 3.70±0.18e | 4.60±0.23b | 4.30±0.21c | 4.50±0.19b | 4.80±0.22a |
| C15:0 | 0.50±0.02a | 0.60±0.02a | 0.50±0.03a | 0.50±0.02a | 0.50±0.03a | 0.50±0.02a | 0.50±0.02a | 0.50±0.03a | 0.50±0.02a | 0.50±0.02a | 0.50±0.03a | 0.50±0.04a |
| C16:0 | 20.90±0.23f | 23.10±0.25a | 22.70±0.22b | 21.50±0.31e | 21.90±0.29d | 22.70±0.27b | 21.40±0.31e | 22.20±0.28cd | 21.30±0.24e | 21.90±0.24d | 22.40±0.31bc | 22.10±0.29cd |
| C17:0 | 0.50±0.03a | 0.50±0.03a | 0.50±0.02a | 0.50±0.04a | 0.50±0.03a | 0.50±0.02a | 0.50±0.04a | 0.50±0.03a | 0.50±0.02a | 0.50±0.03a | 0.50±0.01a | 0.50±0.22a |
| C18:0 | 5.40±0.15bc | 5.80±0.14a | 5.50±0.15abc | 5.30±0.18c | 5.70±0.19ab | 5.70±0.18ab | 5.60±0.18abc | 5.60±0.15abc | 5.50±0.17abc | 5.60±0.21abc | 5.50±0.19abc | 5.80±0.25a |
| C20:0 | 0.60±0.02bc | 0.50±0.03c | 0.60±0.04bc | 0.70±0.03ab | 0.60±0.05bc | 0.70±0.02ab | 0.70±0.03ab | 0.60±0.05bc | 0.70±0.02ab | 0.60±0.02bc | 0.70±0.03ab | 0.80±0.02a |
| C22:0 | 0.20±0.01a | 0.20±0.02a | 0.20±0.01a | 0.20±0.01a | 0.20±0.02a | 0.20±0.01a | 0.20±0.01a | 0.20±0.02a | 0.20±0.01a | 0.20±0.01a | 0.20±0.02a | 0.20±0.01a |
| C24:0 | 0.20±0.01a | 0.20±0.00a | 0.20±0.01a | 0.30±0.02a | 0.30±0.03a | 0.30±0.04a | 0.30±0.02a | 0.30±0.03a | 0.30±0.04a | 0.30±0.02a | 0.30±0.03a | 0.30±0.04a |
| C16:1(n-9) | 6.00±0.21d | 5.50±0.33e | 5.70±0.24e | 6.30±0.30c | 5.70±0.23e | 6.60±0.34b | 6.30±0.30c | 5.70±0.23e | 6.60±0.34b | 6.30±0.35c | 6.40±0.37bc | 6.90±0.31a |
| C18:1(n-9) | 28.2±0.43c | 30.7±0.51a | 29.4±0.43b | 27.8±0.47d | 29.3±0.50b | 27.9±0.49d | 27.80±0.51e | 29.30±0.48b | 27.90±0.61de | 27.40±0.37fg | 28.00±0.42de | 29.20±0.57b |
| C20:1(n-9) | 1.90±0.09a | 1.60±0.08bc | 1.70±0.11ab | 1.70±0.07ab | 1.90±0.11a | 1.90±0.10a | 1.70±0.07ab | 1.90±0.11a | 1.90±0.10a | 1.50±0.12c | 1.60±0.09bc | 1.60±0.08bc |
| C22:1(n-9) | 0.40±0.02a | 0.30±0.03a | 0.40±0.02a | 0.40±0.04a | 0.40±0.03a | 0.40±0.04a | 0.40±0.04a | 0.40±0.03a | 0.40±0.04a | 0.30±0.04a | 0.40±0.03a | 0.40±0.04a |
| C24:1(n-9) | 0.80±0.04a | 0.70±0.02a | 0.60±0.04a | 0.80±0.06a | 0.70±0.02a | 0.70±0.01a | 0.80±0.06a | 0.70±0.02a | 0.70±0.01a | 0.70±0.01a | 0.60±0.03a | 0.70±0.04a |
| C18:2(n-6) | 0.9±0.06a | 0.8±0.03b | 0.9±0.05a | 0.9±0.04a | 0.9±0.05a | 0.9±0.06a | 0.90±0.01a | 0.90±0.02a | 0.90±0.02a | 0.80±0.01a | 0.80±0.01a | 0.90±0.01a |
| C18:3(n-6) | 0.10±0.01a | ND | 0.10±0.02a | 0.10±0.01a | ND | 0.10±0.00a | 0.10±0.01a | ND | 0.10±0.00a | 0.20±0.01a | 0.10±0.01ba | 0.10±0.02a |
| C18:3(n-3) | 0.9±0.05a | 0.8±0.04b | 0.7±0.06c | 0.8±0.02b | 0.8±0.03b | 0.9±0.03a | 0.80±0.01ab | 0.80±0.02ab | 0.90±0.01ab | 0.80±0.02ab | 0.60±0.02b | 0.70±0.01ab |
| C20:2(n-6) | 0.20±0.01a | 0.20±0.02a | 0.10±0.01a | 0.20±0.02a | 0.20±0.01a | 0.20±0.01a | 0.20±0.02a | 0.20±0.01a | 0.20±0.01a | 0.20±0.02a | 0.20±0.00a | 0.20±0.01a |
| C20:4(n-6) | 1.10±0.18bc | 1.20±0.17ab | 1.10±0.10bc | 1.10±0.09bc | 1.10±0.10bc | 1.10±0.08bc | 1.10±0.04bc | 1.10±0.08bc | 1.00±0.19c | 1.20±0.12ab | 1.30±0.14a | 1.10±0.11bc |
| C20:5(n-3) | 7.90±0.47a | 5.90±0.37h | 6.90±0.38e | 7.40±0.47cd | 7.20±0.84d | 6.20±0.68g | 7.80±0.57ab | 6.50±0.49f | 7.70±0.57ab | 7.80±0.34ab | 7.60±0.32bc | 7.40±0.31cd |
| C22:6(n-3) | 17.8±0.26b | 16.90±0.17bc | 16.90±0.24bc | 17.30±0.33abc | 17.50±0.41ab | 17.90±0.19a | 17.10±0.21bc | 17.50±0.51ab | 16.70±0.34c | 17.30±0.24abc | 16.00±0.32d | 14.40±0.52e |
| SFA | 32.10±0.47f | 34.20±0.39bcd | 33.80±0.38cde | 33.30±0.50e | 34.40±0.48abc | 34.20±0.51bcd | 33.50±0.64 | 33.60±0.58de | 33.60±0.47de | 33.90±0.38cde | 34.60±0.61ab | 35.00±0.63a |
| MUFA | 37.30±0.51cd | 38.80±0.43a | 37.80±0.58b | 37.00±0.49e | 36.30±0.39f | 37.20±0.42de | 37.50±0.54c | 38.00±0.68b | 37.50±0.57c | 36.20±0.51f | 37.00±0.57e | 38.80±0.60a |
| PUFA | 28.90±0.65a | 25.80±0.63g | 26.70±0.72f | 28.10±0.87c | 28.10±0.89c | 27.40±0.62d | 28.00±0.95c | 27.00±0.68e | 27.50±0.87d | 28.40±0.58b | 26.70±0.69f | 24.80±0.80h |
| n-9 | 31.30±0.71bcde | 33.30±0.59a | 32.10±0.63abc | 30.60±0.89cde | 30.10±0.91de | 31.60±0.84bcd | 30.70±0.58bcde | 32.30±0.67ab | 30.90±0.84bcde | 29.90±0.98e | 30.60±0.91cde | 31.90±0.87abc |
| n-6 | 2.30±0.12a | 2.20±0.25a | 2.20±0.16a | 2.40±0.15a | 2.40±0.17a | 2.30±0.10a | 2.30±0.21a | 2.20±0.14a | 2.20±0.11a | 2.50±0.14a | 2.50±0.13a | 2.30±0.15a |
| n-3 | 26.60±1.03a | 23.60±0.99g | 24.50±0.87ef | 25.70±1.35b | 25.70±1.3b | 25.10±1.21cd | 25.70±0.98b | 24.80±0.87de | 25.30±0.54c | 25.90±1.24b | 24.20±1.1f | 22.50±1.07k |
| AV | 1.41±0.19e | 1.38±0.12ef | 0.86±0.11i | 1.31±0.11g | 1.65±0.10c | 2.10±0.15a | 1.36±0.21f | 1.41±0.16e | 1.77±0.23b | 1.14±0.11h | 1.48±0.20d | 1.65±0.14c |
| POV | 0.36±0.01k | 0.58±0.01j | 0.57±0.01j | 2.16±0.07c | 1.82±0.01e | 1.7±0.03f | 1.51±0.02g | 0.76±0.04h | 0.69±0.01i | 3.11±0.08a | 2.58±0.02b | 1.93±0.04d |
| TBARS | 0.97±0.04i | 2.32±0.29fjh | 1.83±0.02h | 2.74±0.12efg | 3.64±0.25d | 4.86±0.11c | 2.25±0.17gh | 2.79±0.24ef | 3.61±0.16d | 3.18±0.04de | 14.73±0.62b | 30.88±1.91a |
| HHE | 2.24±0.12i | 0.72±0.08j | 2.37±0.09i | 12.17±1.32d | 9.21±0.41e | 29.69±1.52c | 4.06±0.96gh | 3.05±0.54hi | 6.32±0.62f | 4.75±0.22g | 51.60±4.15b | 86.33±7.54a |
| HNE | 0.22±0.05h | 0.24±0.03h | 0.37±0.03g | 4.65±0.44b | 0.53±0.09e | 1.61±0.04c | 0.25±0.09h | 0.26±0.07h | 0.47±0.09f | 0.87±0.07d | 1.58±0.19c | 5.29±0.54a |

*^a^* C22:6(n-3), Docosahexaenoic Acid(DHA); C20:4(n-6), Arachidonic Acid(ARA); C20:5(n-3), Eicosapentaenoic Acid(EPA); SFA, saturated fatty acid; MUFA, monounsaturated fatty acid; n-9, n-9 fatty acid; PUFA, polyunsaturated fatty acid; n-6, n-6 fatty acid; n-3, n-3 fatty acid; AV, acid value (mg KOH g^-1^); POV, peroxide value (g/100g lipid); TBARS, malondialdehyde (mg/kg fish); HHE, 4-hydroxyhexenal (mg/kg fish); HNE, 4-hydroxynonenal (mg/kg fish). *^b^* Data are mean value of triplicate with SD. *^c^* ND means not detect. Different uppercase denotes significant difference (*P* < 0.05) in the same row.

|  |  | HHE | HNE | MDA | linoleic acid | α-linolenic acid | DHA | EPA | ARA | oleic acid | PUFA | SFA | MUFA | n-3 | n-6 | n-9 | AV | POV |
| --- | --- | --- | --- | --- | --- | --- | --- | --- | --- | --- | --- | --- | --- | --- | --- | --- | --- | --- |
| ·Hot | HHE | 1 | -0.132 | 0.840 | - | - | 0.893 | -0.958 | - | 0.963 | **-0.991*** | 0.216 | 0.765 | **-0.991*** | **-0.991*** | 0.980 | 0.839 | -0.600 |
|  | HNE | -0.132 | 1 | -0.649 | - | - | -0.565 | 0.411 | - | 0.141 | 0.264 | **-0.996*** | 0.537 | 0.264 | 0.264 | 0.067 | -0.650 | 0.872 |
|  | TBARS | 0.840 | -0.649 | 1 | - | - | **0.994*** | -0.960 | - | 0.661 | -0.905 | 0.712 | 0.293 | -0.905 | -0.905 | 0.716 | **1.000**** | -0.938 |
| ·Cold | HHE | 1 | 0.952 | 0.761 | 0.954 | - | -0.976 | 0.688 | -0.954 | -0.695 | 0.299 | 0.218 | -0.736 | 0.360 | -0.218 | -0.654 | 0.918 | -0.296 |
|  | HNE | 0.952 | 1 | 0.924 | **1.000**** | - | -0.862 | 0.432 | **-1.000**** | -0.440 | -0.008 | 0.507 | -0.493 | 0.056 | -0.507 | -0.390 | **0.996*** | -0.575 |
|  | TBARS | 0.761 | 0.924 | 1 | 0.920 | - | -0.602 | 0.053 | -0.920 | -0.062 | -0.391 | 0.799 | -0.122 | -0.331 | -0.799 | -0.007 | 0.956 | -0.845 |
| ·Natural | HHE | 1 | 0.897 | 0.984 | -0.572 | 0.820 | **-0.989*** | **-0.996*** | -0.424 | 0.962 | **-0.993*** | **0.998*** | 0.954 | **-0.996*** | -0.820 | 0.967 | **0.995*** | **-0.990*** |
|  | HNE | 0.897 | 1 | 0.962 | -0.150 | **0.989*** | -0.951 | -0.931 | -0.781 | 0.983 | -0.942 | 0.863 | **0.988*** | -0.931 | **-0.989*** | 0.980 | 0.846 | -0.949 |
|  | TBARS | 0.984 | 0.962 | 1 | -0.415 | 0.910 | **-0.999*** | **-0.995*** | -0.580 | **0.996*** | **-0.998*** | 0.968 | **0.992*** | **-0.995*** | -0.910 | **0.997*** | 0.959 | **-0.999*** |

**Table S2. Pearson correlation coefficients between the levels of TBARS, HHE, HNE and other oxidation indices** *^a^*

*^a^* Numbers in bold indicate significant correlations. *^b^* * Significance at *P* < 0.05. *^c^* ** Significance at *P* < 0.01.

**
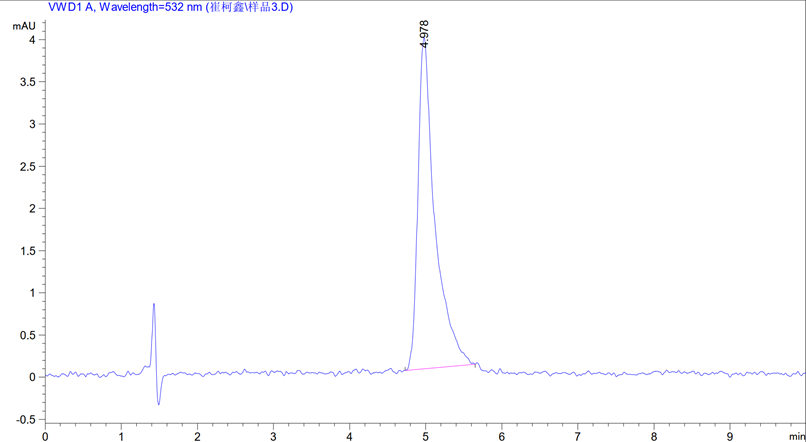
**

**Figure S1.** Representative HPLC chromatograms of TBARS


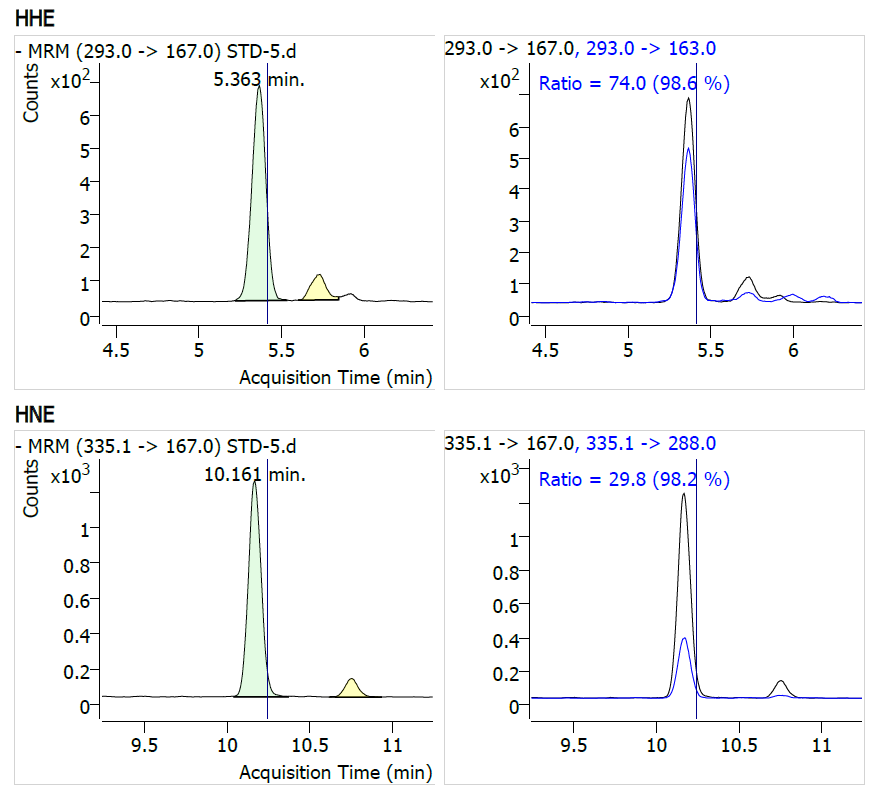
HHE

**M-1**

**L-1**

**K-1**

**J-1**

**I-1**

**H-1**

**G-1**

**F-1**

**E-1**

**D-1**

**C-1**

**B-1**

**A-1**


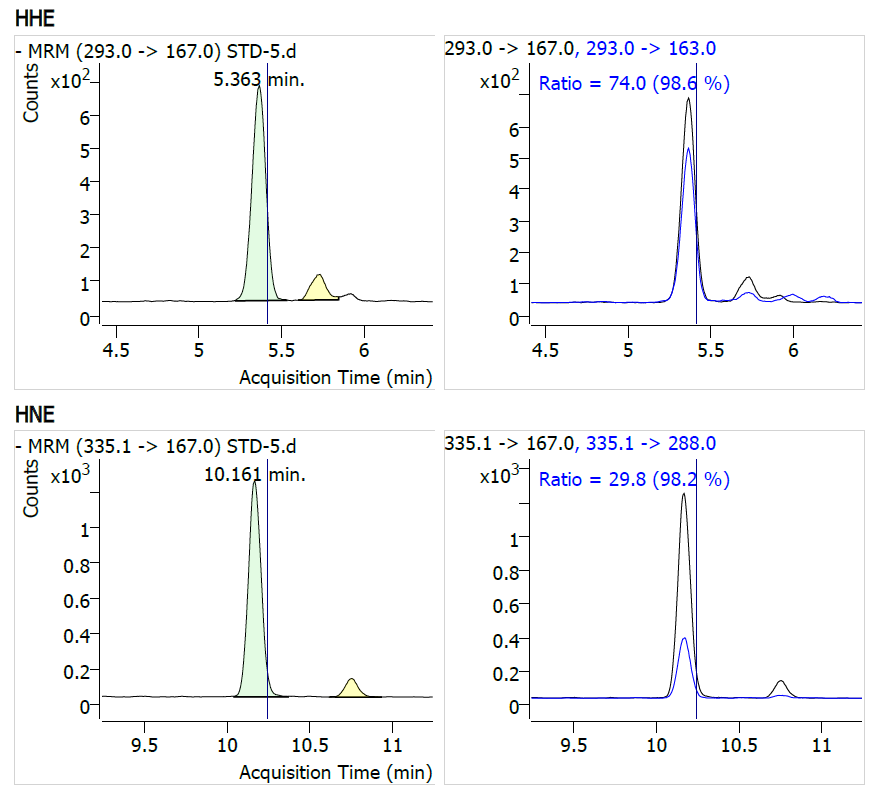
HNE

**B-2**

**M-2**

**K-2**

**J-2**

**I-2**

**H-2**

**G-2**

**F-2**

**E-2**

**D-2**

**A-2**

**L-2**

**C-2**

**Figure S2.** Alkenal chromatographic mass spectrometry information.

(**A-1**: 100ppb of HHE-DNPH and HHE-d_3_-DNPH; **A-2**: 100ppb of HNE-DNPH and HNE-d_3_-DNPH; **B-1**: HHE chromatograms of raw material; **B-2**: HNE chromatograms of raw material; **C-1**: HHE chromatograms of curing samples; **C-2**: HNE chromatograms of curing samples; **D-1**: HHE chromatograms of desalting samples; **D-2**: HNE chromatograms of desalting samples; **E-1**: HHE chromatograms of early-stage in hot air-drying process; **E-2**: HNE chromatograms of early-stage in hot air-drying process; **F-1**: HHE chromatograms of mid-stage in hot air-drying process; **F-2**: HNE chromatograms of mid-stage in hot air-drying process; **G-1**: HHE chromatograms of end-stage in hot air-drying process; **G-2**: HNE chromatograms of end-stage in hot air-drying process; **H-1**: HHE chromatograms of early-stage in cold air-drying process; **H-2**: HNE chromatograms of early-stage in cold air-drying process; **I-1**: HHE chromatograms of mid-stage in cold air-drying process; **I-2**: HNE chromatograms of mid-stage in cold air-drying process; **J-1**: HHE chromatograms of end-stage in cold air-drying process; **J-2**: HNE chromatograms of end-stage in cold air-drying process; **K-1**: HHE chromatograms of early-stage in sun drying process; **K-2**: HNE chromatograms of early-stage in sun drying process; **L-1**: HHE chromatograms of mid-stage in sun drying process; **L-2**: HNE chromatograms of mid-stage in sun drying process; **M-1**: HHE chromatograms of end-stage in sun drying process; **M-2**: HNE chromatograms of end-stage in sun drying process. )


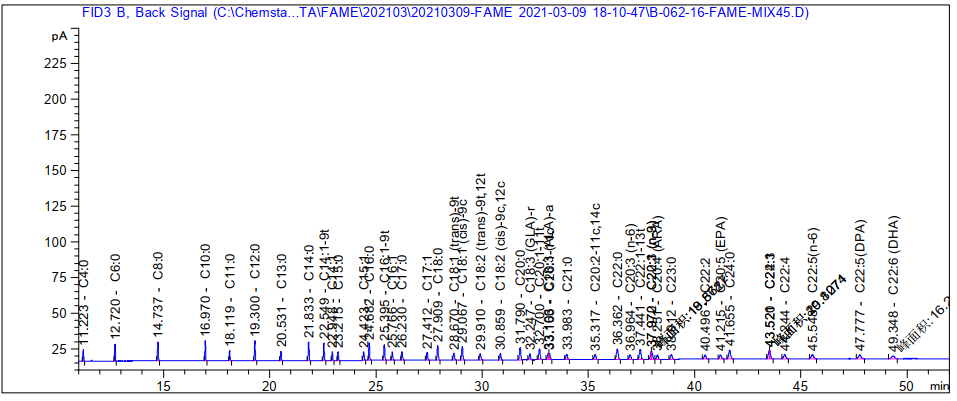

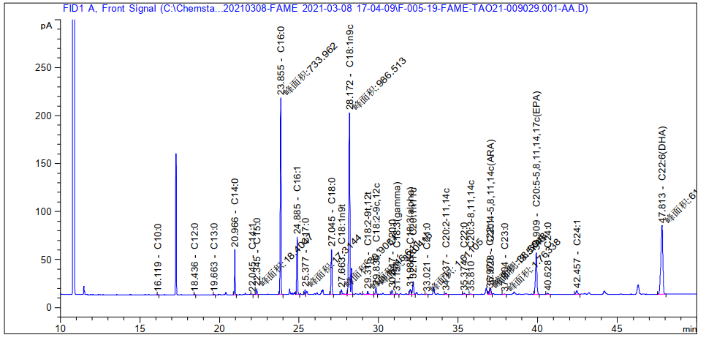

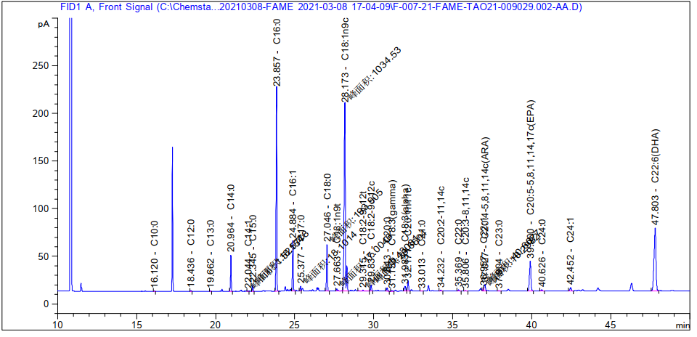

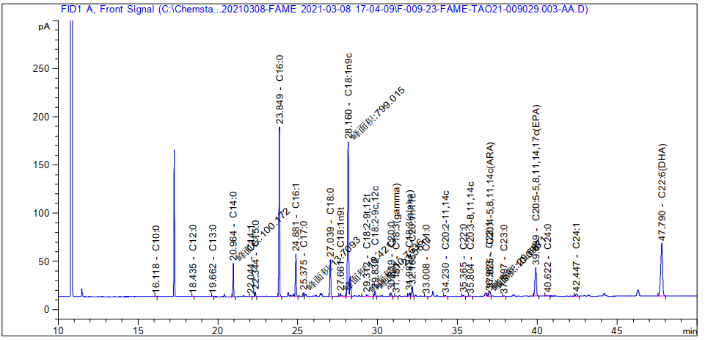

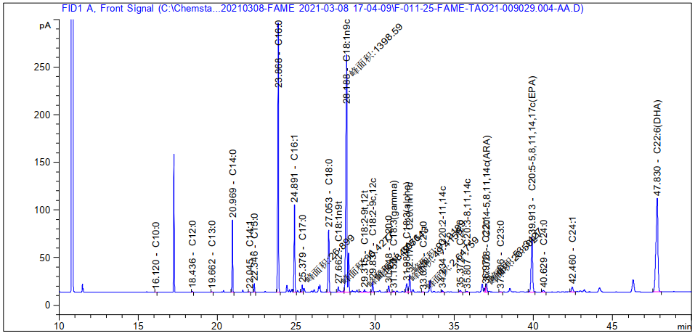

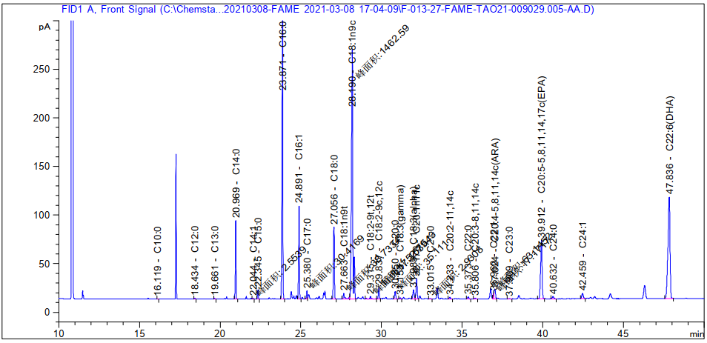

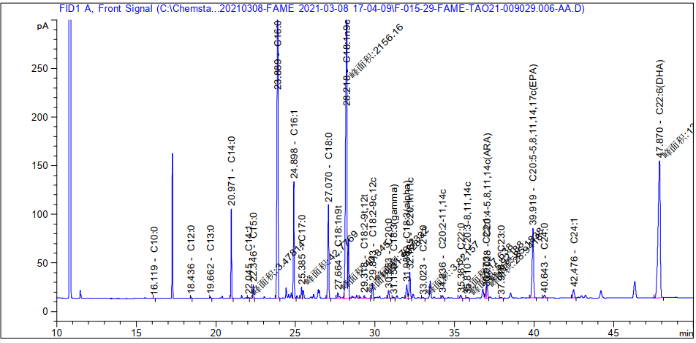

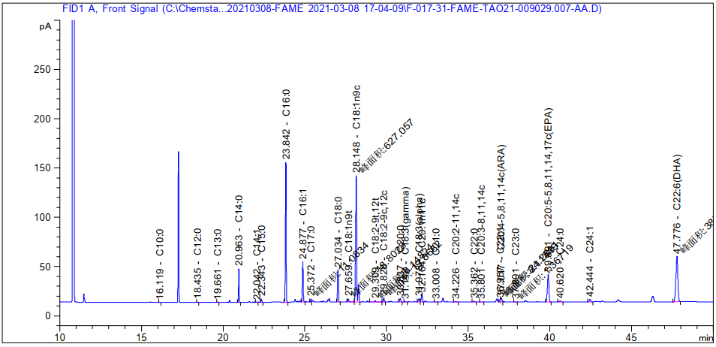

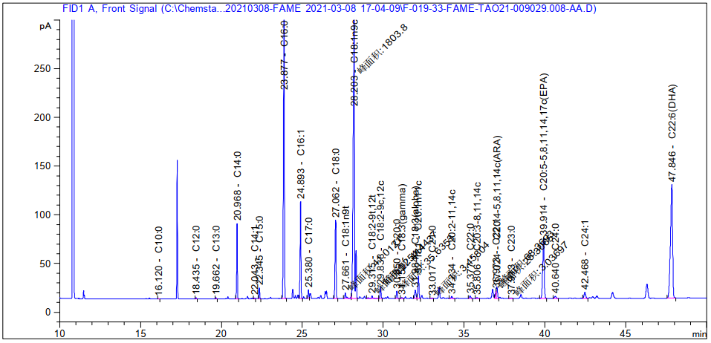


**I**

**A**

**B**

**C**

**D**

**E**

**F**

**G**

**H**


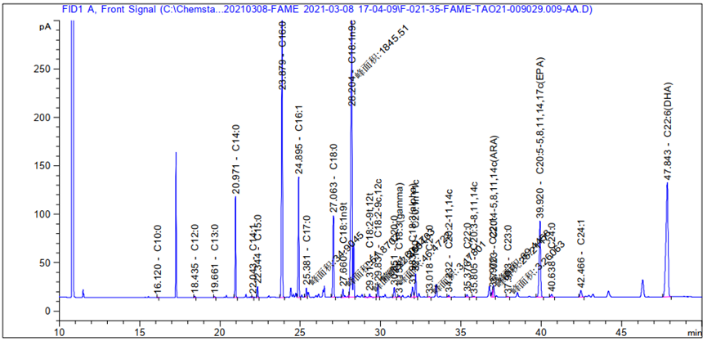

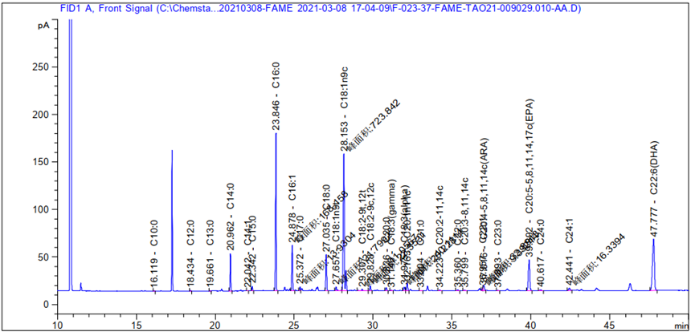

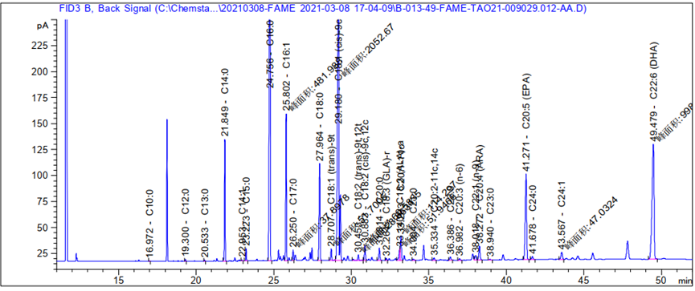

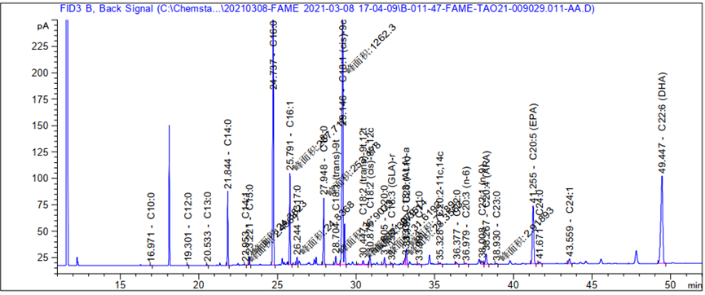


**K**

**J**

**M**

**L**

**Figure S3. Fatty acid** **GC spectrums.**

(**A**: GC spectrums of fatty acids aflatoxin mixture; **B**: GC spectrums of raw material; **C**: GC spectrums of curing samples; **D**: GC spectrums of desalting samples; **E**: GC spectrums of early-stage in hot air-drying process; **F**: GC spectrums of mid-stage in hot air-drying process; **G**: GC spectrums of end-stage in hot air-drying process; **H**: GC spectrums of early-stage in cold air-drying process; **I**: GC spectrums of mid-stage in cold air-drying process; **J**: GC spectrums of end-stage in cold air-drying process; **K**: GC spectrums of early-stage in sun drying process; **L**: GC spectrums of mid-stage in sun drying process; **M**: GC spectrums of end-stage in sun drying process.)
